# The aluminoarsenate K_1.8_Sr_0.6_Al_3_(AsO_4_)_4_

**DOI:** 10.1107/S1600536812014304

**Published:** 2012-04-13

**Authors:** Anissa Haj Abdallah, Amor Haddad

**Affiliations:** a Institut Supérieur des Sciences Appliquées et Technologie de Gabès, Avenue Omar Ibn El Khattab, 6072 Zrig, Gabès, Tunisia; b Laboratoire de Matériaux et Cristallochime, Institut Supérieur des Sciences Appliquées et Technologie de Mahdia, Avenue El Mourouj, Sidi Messoud 5111 Hiboun, Mahdia, Tunisia

## Abstract

The title compound, potassium strontium trialuminium tetra­arsenate, was prepared by solid-state reaction. The structure consists of AlO_6_ octa­hedra (site symmetries 2.. and 2/*m*) and two AsO_4_ tetra­hedra (.2. and *m*..) sharing corners and edges to form a two-dimensional structure parallel to (010). The cations are occupationally disordered and are located in the interlayer space. For both types of cations, distorted coordination polyhedra are observed.

## Related literature

For further information on this structure type, see: K_3_Cr_3_(AsO_4_)_4_ (Friaa *et al.*, 2003 ▶); K_3_Fe_3_(AsO_4_)_4_ (Ouerfelli *et al.*, 2005 ▶). For similar structures, see: K_3_Fe_3_(PO_4_)_4_·H_2_O (Lii, 1995 ▶); Na_3_Fe_3_(PO_4_)_4_ (Lajmi *et al.*, 2002 ▶). For background to the bond-valence method, see: Brown & Altermatt (1985 ▶).

## Experimental

### 

#### Crystal data

K_1.8_Sr_0.6_Al_3_(AsO_4_)_4_
*M* *_r_* = 759.57
Orthorhombic, 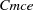

*a* = 10.567 (3) Å
*b* = 20.531 (4) Å
*c* = 6.388 (1) Å
*V* = 1385.9 (5) Å^3^
*Z* = 4
Mo *K*α radiation
μ = 12.67 mm^−1^
*T* = 293 K
0.36 × 0.22 × 0.14 mm

#### Data collection

Enraf–Nonius CAD-4 diffractometer
Absorption correction: ψ scan (North *et al.*, 1968 ▶) *T*_min_ = 0.088, *T*_max_ = 0.292
1510 measured reflections
796 independent reflections
704 reflections with *I* > 2σ(*I*)
*R*_int_ = 0.024
2 standard reflections every 120 min intensity decay: 0.4%

#### Refinement

*R*[*F* ^2^ > 2σ(*F* ^2^)] = 0.039
*wR*(*F* ^2^) = 0.112
*S* = 1.09
796 reflections
88 parameters
Δρ_max_ = 1.68 e Å^−3^
Δρ_min_ = −1.45 e Å^−3^

### 

Data collection: *CAD-4 EXPRESS* (Enraf–Nonius, 1994 ▶); cell refinement: *CAD-4 EXPRESS*; data reduction: *XCAD4* (Harms & Wocadlo, 1995 ▶); program(s) used to solve structure: *SHELXS97* (Sheldrick, 2008 ▶); program(s) used to refine structure: *SHELXL97* (Sheldrick, 2008 ▶); molecular graphics: *DIAMOND* (Brandenburg, 1998 ▶); software used to prepare material for publication: *WinGX* (Farrugia, 1999 ▶).

## Supplementary Material

Crystal structure: contains datablock(s) I, global. DOI: 10.1107/S1600536812014304/br2195sup1.cif

Structure factors: contains datablock(s) I. DOI: 10.1107/S1600536812014304/br2195Isup2.hkl

Supplementary material file. DOI: 10.1107/S1600536812014304/br2195Isup3.cml

Additional supplementary materials:  crystallographic information; 3D view; checkCIF report

## Figures and Tables

**Fig. 1.**
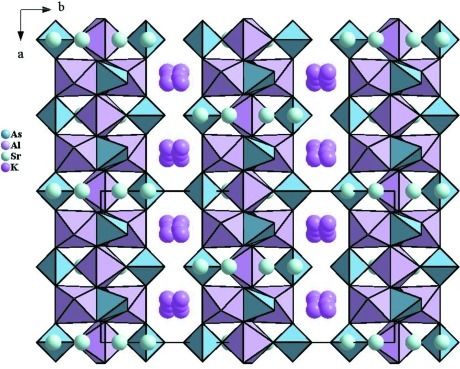
: Projection of the structure of K1.8Sr0.6Al3(AsO4)4 along the c axis.

**Fig. 2.**
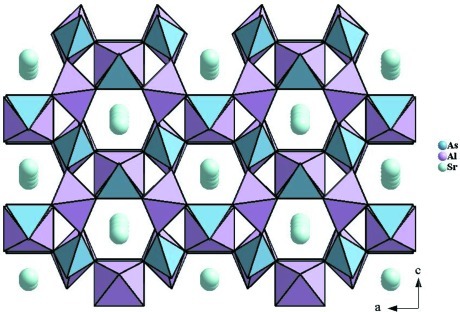
: A view of a layer showing the windows.

**Fig. 3.**
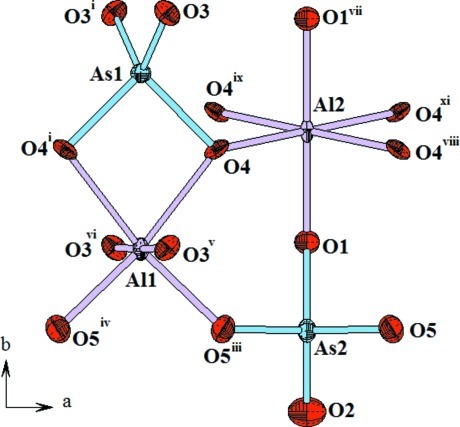
: The asymmetric unit of the structure of K1.8Sr0.6Al3(AsO4)4.

## supplementary crystallographic information

### Comment

The crystal structure of K_1.8_Sr_0.6_Al_3_(AsO_4_)_4_ is a two-dimensional network formed by curved layers perpendicular to the *b* axis. Each layer consists of AlO_6_ octahedra and AsO_4_ tetrahedra sharing corners and edges (Fig. 1). This structural arrangement leads to six-membered windows within the layer (Fig. 2). It is isostructural with the compounds K_3_Cr_3_(AsO_4_)_4_ (Friaa *et al.*, 2003) and K_3_Fe_3_(AsO_4_)_4_ (Ouerfelli *et al.*, 2005). The asymmetric unit is the link-up of two AlO_6_ octahedra and As2O_4_ tetrahedron by corners and As1O_4_ tetrahedron by edges (Fig. 3). The common edge O4···O4, is the shortest oxygen distance. The As1O_4_ tetrahedron lies on two fold axis and is distorted. It has two type of As—O bonds, two short distances and two long distances involving the bridging oxygen atoms O4. The As2O_4_ tetrahedron is located on mirror plane. It shares two oxygen atoms O5 with two equivalent Al1O_6_ octahedra and one oxygen atom O1, which is situated on mirror plane, with Al2O_6_ octahedron. The fourth oxygen atom O2 which lies on mirror plane is not coordinated and points to the interlayer space. The Al1O_6_ octahedron is strongly distorted which the O4—Al1—O4 angle 75.6 ° is more acute than those according to an ideal octahedron. The mean distance of Al1—O 1.930 (4) Å. The Al2O_6_ octahedron which lies on a crystallographic center of symmetry is less distorted than the Al1O_6_ octahedron which lies on a two fold axis. The structure is characterized by two cationic sites. The site (0, 1/2, 1/2) located in the interlayer space and the site (1/4, 1/2, 1/4) confined in windows. The difference to be indicated is the cationic distribution in the similar structure compounds. Both strontium cations Sr1 and Sr2 occupy the windows and the potassium cations K1 and K2 are located in interlayers space of the structure. The bond valence sum of the K1, K2 and Sr1 are in a good agreement with their oxidation states (Brown & Altermatt, 1985). But for the Sr2, the bond strength is higher than the expected value +2 suggesting that the SrO_9_ appear too small for this ion which is acceptable given the small number of Sr atoms that occupy this site. The valence sums around the Al atoms are both low (2.6 vu) but attempts to introduce other elements onto these sites were not successfull. The resulting difference electron density contains several large features reflecting the difficulty in determining the precise locations of Sr and and K.

### Experimental

The purpose is to obtain a phase from the molars proportions of (2:0.5:4) of a mixture of KNO_3_, Sr(NO_3_)_2_ and NH_4_H_2_AsO_4_. The mixture was finely ground and calcined at 723 K in a porcelain crucible. Then the temperature was held at 943 K during 26 h. A slow cooling in a speed of 2 K/h until 893 K and of 5 K/h until 823 K was proceeded. The fusion was reached. A long wash in the boiling water allowed us to isolate some parallelepipedic colourless crystals with acceptable size for an analysis by X-ray diffraction on single-crystal. The qualitative analysis by electron microscope probe of a selected crystal revealed the presence of aluminium atom which comes from the crucible, as well as the different elements of the compound composition.

### Refinement

The localization of the strontium and potassium atoms is delicate because of the existence of disorder. The Fourier difference synthesis reveals an intense peak and three nearby peaks relatively less intense. It was then necessary to take considering the peaks which could have a structural meaning because of their environments in atoms of oxygen. A constraint, respecting the electroneutrality, was applied to the occupation rates of the cations. Furthermore, the refinement anisotropy leading to very deformed ellipsoids, the condition EADP allowed by the program SHELX was applied. The high value of the electron densities occurs at 1.68 e/A^3^ from heavy atom As1 is due to the effects of Fourier series termination.

### Crystal data

**Table:**

K_1.8_Sr_0.6_Al_3_(AsO_4_)_4_	*F*(000) = 1424
*M**_r_* = 759.57	*D*_x_ = 3.641 Mg m^−^^3^
Orthorhombic, *C**m**c**e*	Mo *K*α radiation, λ = 0.71073 Å
Hall symbol: -C 2bc 2	Cell parameters from 25 reflections
*a* = 10.567 (3) Å	θ = 2.2–27°
*b* = 20.531 (4) Å	µ = 12.67 mm^−^^1^
*c* = 6.388 (1) Å	*T* = 293 K
*V* = 1385.9 (5) Å^3^	Parallelipedic, colourless
*Z* = 4	0.36 × 0.22 × 0.14 mm

### Data collection

**Table:**

Enraf–Nonius CAD-4 diffractometer	704 reflections with *I* > 2σ(*I*)
Radiation source: fine-focus sealed tube	*R*_int_ = 0.024
Graphite monochromator	θ_max_ = 27.0°, θ_min_ = 3.8°
ω/2θ scans	*h* = −13→0
Absorption correction: ψ scan (North *et al.*, 1968)	*k* = −26→0
*T*_min_ = 0.088, *T*_max_ = 0.292	*l* = −8→8
1510 measured reflections	2 standard reflections every 120 min
796 independent reflections	intensity decay: 0.4%

### Refinement

**Table:**

Refinement on *F*^2^	Primary atom site location: real-space vector search
Least-squares matrix: full	Secondary atom site location: difference Fourier map
*R*[*F*^2^ > 2σ(*F*^2^)] = 0.039	*w* = 1/[σ^2^(*F*_o_^2^) + (0.0826*P*)^2^] where *P* = (*F*_o_^2^ + 2*F*_c_^2^)/3
*wR*(*F*^2^) = 0.112	(Δ/σ)_max_ < 0.001
*S* = 1.09	Δρ_max_ = 1.68 e Å^−^^3^
796 reflections	Δρ_min_ = −1.45 e Å^−^^3^
88 parameters	Extinction correction: *SHELXL97* (Sheldrick, 2008), Fc^*^=kFc[1+0.001xFc^2^λ^3^/sin(2θ)]^-1/4^
0 restraints	Extinction coefficient: 0.0006 (3)

### Special details

**Table:**

Geometry. All e.s.d.'s (except the e.s.d. in the dihedral angle between two l.s. planes) are estimated using the full covariance matrix. The cell e.s.d.'s are taken into account individually in the estimation of e.s.d.'s in distances, angles and torsion angles; correlations between e.s.d.'s in cell parameters are only used when they are defined by crystal symmetry. An approximate (isotropic) treatment of cell e.s.d.'s is used for estimating e.s.d.'s involving l.s. planes.
Refinement. Refinement of *F*^2^ against ALL reflections. The weighted *R*-factor *wR* and goodness of fit *S* are based on *F*^2^, conventional *R*-factors *R* are based on *F*, with *F* set to zero for negative *F*^2^. The threshold expression of *F*^2^ > σ(*F*^2^) is used only for calculating *R*-factors(gt) *etc*. and is not relevant to the choice of reflections for refinement. *R*-factors based on *F*^2^ are statistically about twice as large as those based on *F*, and *R*- factors based on ALL data will be even larger.

### Fractional atomic coordinates and isotropic or equivalent isotropic displacement parameters (Å^2^)

**Table:**

	*x*	*y*	*z*	*U*_iso_*/*U*_eq_	Occ. (<1)
As1	0.7500	0.54444 (4)	0.2500	0.0058 (3)	
As2	0.0000	0.65488 (4)	−0.05958 (11)	0.0070 (3)	
Al1	0.7500	0.40809 (11)	0.2500	0.0054 (5)	
Al2	0.0000	0.5000	0.0000	0.0069 (6)	
Sr1	0.010 (3)	0.6619 (4)	0.4463 (8)	0.056 (4)	0.12 (2)
Sr2	0.006 (8)	0.5644 (17)	0.477 (4)	0.056 (4)	0.03 (2)
K1	0.2111 (8)	0.7265 (4)	0.3993 (12)	0.062 (2)	0.33 (1)
K2	0.258 (2)	0.7305 (10)	0.190 (3)	0.062 (2)	0.12 (1)
O1	0.0000	0.5866 (3)	0.0858 (7)	0.0090 (10)	
O2	0.0000	0.7176 (3)	0.1009 (9)	0.0180 (12)	
O3	0.7104 (3)	0.59095 (18)	0.0477 (5)	0.0092 (7)	
O4	0.6367 (3)	0.48557 (16)	0.2962 (5)	0.0078 (7)	
O5	−0.1259 (3)	0.65351 (18)	−0.2222 (5)	0.0120 (8)	

### Atomic displacement parameters (Å^2^)

**Table:**

	*U*^11^	*U*^22^	*U*^33^	*U*^12^	*U*^13^	*U*^23^
As1	0.0061 (4)	0.0055 (4)	0.0057 (4)	0.000	0.0008 (2)	0.000
As2	0.0047 (4)	0.0081 (4)	0.0083 (4)	0.000	0.000	−0.0014 (2)
Al1	0.0042 (10)	0.0075 (11)	0.0046 (9)	0.000	0.0008 (6)	0.000
Al2	0.0026 (13)	0.0090 (15)	0.0088 (13)	0.000	0.000	0.0000 (11)
Sr1	0.058 (7)	0.074 (5)	0.034 (2)	0.038 (9)	−0.004 (5)	0.003 (2)
Sr2	0.058 (7)	0.074 (5)	0.034 (2)	0.038 (9)	−0.004 (5)	0.003 (2)
K1	0.074 (5)	0.051 (3)	0.060 (5)	0.037 (4)	−0.002 (4)	0.011 (3)
K2	0.074 (5)	0.051 (3)	0.060 (5)	0.037 (4)	−0.002 (4)	0.011 (3)
O1	0.008 (2)	0.008 (2)	0.010 (2)	0.000	0.000	0.0038 (19)
O2	0.022 (3)	0.014 (3)	0.018 (3)	0.000	0.000	−0.004 (2)
O3	0.0094 (17)	0.0098 (18)	0.0084 (15)	0.0029 (15)	−0.0011 (13)	0.0003 (13)
O4	0.0086 (16)	0.0060 (16)	0.0088 (15)	−0.0033 (15)	0.0017 (14)	0.0011 (11)
O5	0.0089 (18)	0.0115 (17)	0.0155 (18)	−0.0023 (16)	−0.0024 (14)	0.0051 (13)

### Geometric parameters (Å, º)

**Table:**

As1—O3^i^	1.661 (3)	Sr1—O2	2.489 (8)
As1—O3	1.661 (3)	Sr1—O5^xiii^	2.565 (17)
As1—O4^i^	1.727 (3)	Sr1—O3^ix^	2.57 (2)
As1—O4	1.727 (3)	Sr1—O2^xiv^	2.666 (10)
As2—O2	1.646 (6)	Sr1—O3^xi^	2.75 (3)
As2—O1	1.682 (5)	Sr1—O1	2.775 (9)
As2—O5^ii^	1.688 (3)	Sr2—O3^ix^	2.23 (9)
As2—O5	1.688 (3)	Sr2—O4^xv^	2.67 (4)
Al1—O5^iii^	1.831 (4)	Sr2—O4^ix^	2.75 (4)
Al1—O5^iv^	1.831 (4)	Sr2—O1	2.54 (2)
Al1—O3^v^	1.947 (3)	Sr2—O5^xii^	2.94 (5)
Al1—O3^vi^	1.947 (3)	Sr2—O3^xi^	2.36 (8)
Al1—O4^i^	2.013 (4)	K1—O5^xiv^	2.735 (7)
Al1—O4	2.013 (4)	K1—O3^ix^	2.804 (8)
Al2—O1^vii^	1.861 (5)	K1—O2^xiv^	2.819 (8)
Al2—O1	1.861 (5)	K1—O2	2.940 (9)
Al2—O4^viii^	1.967 (3)	K2—O5^xvi^	2.69 (2)
Al2—O4^ix^	1.967 (3)	K2—O2	2.80 (3)
Al2—O4^x^	1.967 (3)	K2—O5^xiv^	2.817 (19)
Al2—O4^xi^	1.967 (3)	K2—O2^xvii^	2.90 (2)
Sr1—O5^xii^	2.450 (15)	K2—O3^xviii^	3.02 (2)
			
O3^i^—As1—O3	109.8 (2)	O5^xiii^—Sr1—O3^ix^	114.3 (3)
O3^i^—As1—O4^i^	111.16 (17)	O5^xii^—Sr1—O2^xiv^	76.4 (3)
O3—As1—O4^i^	116.37 (16)	O2—Sr1—O2^xiv^	84.2 (2)
O3^i^—As1—O4	116.37 (16)	O5^xiii^—Sr1—O2^xiv^	74.6 (3)
O3—As1—O4	111.16 (17)	O3^ix^—Sr1—O2^xiv^	123.7 (9)
O4^i^—As1—O4	91.2 (2)	O5^xii^—Sr1—O3^xi^	112.0 (5)
O2—As2—O1	108.0 (3)	O2—Sr1—O3^xi^	102.6 (7)
O2—As2—O5^ii^	113.34 (17)	O5^xiii^—Sr1—O3^xi^	58.5 (5)
O1—As2—O5^ii^	109.03 (16)	O3^ix^—Sr1—O3^xi^	113.5 (3)
O2—As2—O5	113.34 (17)	O2^xiv^—Sr1—O3^xi^	116.9 (8)
O1—As2—O5	109.03 (16)	O5^xii^—Sr1—O1	134.2 (6)
O5^ii^—As2—O5	104.0 (2)	O2—Sr1—O1	61.2 (2)
O5^iii^—Al1—O5^iv^	92.6 (2)	O5^xiii^—Sr1—O1	128.7 (9)
O5^iii^—Al1—O3^v^	93.80 (16)	O3^ix^—Sr1—O1	74.3 (3)
O5^iv^—Al1—O3^v^	87.00 (16)	O2^xiv^—Sr1—O1	145.3 (3)
O5^iii^—Al1—O3^vi^	87.00 (16)	O3^xi^—Sr1—O1	71.6 (4)
O5^iv^—Al1—O3^vi^	93.80 (16)	O3^ix^—Sr2—O4^xv^	69 (2)
O3^v^—Al1—O3^vi^	178.8 (3)	O3^ix^—Sr2—O4^ix^	67.4 (18)
O5^iii^—Al1—O4^i^	96.06 (14)	O4^xv^—Sr2—O4^ix^	90.0 (17)
O5^iv^—Al1—O4^i^	170.55 (17)	O3^ix^—Sr2—O1	85.0 (19)
O3^v^—Al1—O4^i^	88.66 (15)	O4^xv^—Sr2—O1	147 (3)
O3^vi^—Al1—O4^i^	90.43 (15)	O4^ix^—Sr2—O1	59.7 (7)
O5^iii^—Al1—O4	170.55 (17)	O4^xv^—Sr2—O5^xii^	61.2 (11)
O5^iv^—Al1—O4	96.06 (14)	O4^ix^—Sr2—O5^xii^	125 (3)
O3^v^—Al1—O4	90.43 (15)	O1—Sr2—O5^xii^	123 (2)
O3^vi^—Al1—O4	88.66 (15)	O3^ix^—Sr2—O3^xi^	151.3 (17)
O4^i^—Al1—O4	75.6 (2)	O4^xv^—Sr2—O3^xi^	130 (2)
O1^vii^—Al2—O1	180.0 (3)	O4^ix^—Sr2—O3^xi^	125 (2)
O1^vii^—Al2—O4^viii^	87.08 (14)	O1—Sr2—O3^xi^	82.4 (17)
O1—Al2—O4^viii^	92.92 (14)	O5^xii^—Sr2—O3^xi^	108.5 (13)
O1^vii^—Al2—O4^ix^	92.92 (14)	O5^xiv^—K1—O3^ix^	158.0 (4)
O1—Al2—O4^ix^	87.08 (14)	O5^xiv^—K1—O2^xiv^	60.2 (2)
O4^viii^—Al2—O4^ix^	85.5 (2)	O3^ix^—K1—O2^xiv^	110.3 (3)
O1^vii^—Al2—O4^x^	87.08 (14)	O5^xiv^—K1—O2	67.8 (2)
O1—Al2—O4^x^	92.92 (14)	O3^ix^—K1—O2	90.8 (3)
O4^viii^—Al2—O4^x^	94.5 (2)	O2^xiv^—K1—O2	73.8 (2)
O4^ix^—Al2—O4^x^	180.0	O5^xvi^—K2—O2	123.0 (7)
O1^vii^—Al2—O4^xi^	92.92 (14)	O5^xvi^—K2—O5^xiv^	57.4 (4)
O1—Al2—O4^xi^	87.08 (14)	O2—K2—O5^xiv^	68.8 (5)
O4^viii^—Al2—O4^xi^	180.00 (19)	O5^xvi^—K2—O2^xvii^	69.0 (6)
O4^ix^—Al2—O4^xi^	94.5 (2)	O2—K2—O2^xvii^	160.9 (8)
O4^x^—Al2—O4^xi^	85.5 (2)	O5^xiv^—K2—O2^xvii^	114.9 (8)
O5^xii^—Sr1—O2	145.1 (10)	O5^xvi^—K2—O3^xviii^	144.3 (9)
O5^xii^—Sr1—O5^xiii^	64.0 (2)	O2—K2—O3^xviii^	87.6 (7)
O2—Sr1—O5^xiii^	137.6 (9)	O5^xiv^—K2—O3^xviii^	156.4 (10)
O5^xii^—Sr1—O3^ix^	62.4 (5)	O2^xvii^—K2—O3^xviii^	87.4 (5)
O2—Sr1—O3^ix^	108.1 (7)		

Symmetry codes: (i) −*x*+3/2, *y*, −*z*+1/2; (ii) −*x*, *y*, *z*; (iii) *x*+1, −*y*+1, −*z*; (iv) −*x*+1/2, −*y*+1, *z*+1/2; (v) *x*, −*y*+1, −*z*; (vi) −*x*+3/2, −*y*+1, *z*+1/2; (vii) −*x*, −*y*+1, −*z*; (viii) *x*−1/2, −*y*+1, *z*−1/2; (ix) *x*−1/2, *y*, −*z*+1/2; (x) −*x*+1/2, −*y*+1, *z*−1/2; (xi) −*x*+1/2, *y*, −*z*+1/2; (xii) −*x*, *y*, *z*+1; (xiii) *x*, *y*, *z*+1; (xiv) −*x*, −*y*+3/2, *z*+1/2; (xv) *x*−1/2, −*y*+1, *z*+1/2; (xvi) *x*+1/2, −*y*+3/2, −*z*; (xvii) *x*+1/2, *y*, −*z*+1/2; (xviii) −*x*+1, *y*, *z*.
